# Effect of short-term orthokeratology lens or ordinary frame glasses wear on corneal thickness, corneal endothelial cells and vision correction in adolescents with low to moderate myopia

**DOI:** 10.1186/s12886-019-1222-y

**Published:** 2019-11-28

**Authors:** Shuyi Yuan, Shuxian Zhang, Yanglin Jiang, Lihua Li

**Affiliations:** Tianjin Eye Hospital, Tianjin Key Lab of Ophthalmology and Visual Science, Tianjin Eye Institute, No.4 Gansu Road, Heping District, Tianjin City, 300022 China

**Keywords:** Orthokeratology lens, Frame glasses, Correction of myopia, Corneal thickness, Corneal endothelial cells

## Abstract

**Background:**

We aimed to investigate the effect of short-term orthokeratology lens or frame glasses wear on corneal thickness, corneal endothelial cells and vision correction in adolescents with low to moderate myopia.

**Methods:**

Data of 100 adolescents with low to moderate myopia were retrospectively analyzed. The patients were assigned into two groups. The experimental group were treated with night-wear orthokeratology lens, and control group were treated with ordinary frame glasses. Follow up was carried out at the 3rd, 6th and 12th months of treatment. The naked-eye vision, diopter, corneal curvature, intraocular pressure, axial length, endothelial cell count and central corneal thickness were examined. Complications within 12 months were observed, and corneal fluorescein staining was graded.

**Results:**

The naked-eye vision of the experimental group was significantly higher than that of the control group at the 3rd, 6th and 12th months, while the diopter of the experimental group was significantly lower than that of the control group at these time points. The corneal curvature of the experimental group was significantly decreased when comparing with that of the control group at the above 3 time points. The increase of axial length in the experimental group was significantly less than that in the control group at the 6th and 12th months.

**Conclusions:**

Short-term orthokeratology lens wear can effectively improve the naked-eye vision in adolescents with low to moderate myopia without significant impact on the central corneal thickness and corneal endothelial cells. It is a relatively safe method to correct myopia.

## Background

Myopia refers to a pathological phenomenon that when parallel light passes through the dioptric system (the body is completely relaxed) and the focal point is located in front of the retina, so patients with myopia see distant blurred and near clear [1]. At present, although there are many studies on myopia, its pathogenesis is still unclear. Most studies indicate that myopia is related to genetic and environmental factors [2]. Myopia, as a common disease among adolescents, has an increasing incidence and a trend toward the younger [3]. Statistics showed that the number of myopic patients in the world had reached 1.406 billion in 2000, and nearly 2 billion in 2010, accounting for about 30% of the global population. It was predicted that the number of myopic patients worldwide might exceed 4.7 billion by 2050, accounting for 50% of the global population [4]. Various long-term complications might occur in patients with the worsen of myopia, including cataract, glaucoma or even retinal detachment, which seriously impact the visual function of patients [5]. Research statistics showed that more than 300,000 people in China were blind due to myopia [6]. Therefore, the treatment for myopia is one of the major clinical issues. 

The approaches to correct myopia in clinic include drug therapy, surgery, frame glasses and corneal contact lenses etc. [7]. The most common way of correction is using a pair of frame glasses for it is a simple method and the glasses can be changed at will according to the change of the patient’s eyesight [8]. Drug treatment can be divided into long-term and short-term treatment. At present, low-concentration atropine has made favorable progress in controlling myopia, however, long-term use of drugs, which results side effects and drug resistance, needs to be further improved [9]. Surgical treatment is generally used in patients over 18 years and with stable diopters, which is not recommended for adolescents and children [10]. Corneal contact lens consists of a plurality of arc segments and includes three types: soft lens, rigid lens and orthokeratology (OK) lens. Among them, the OK lens plays a significant role against myopia and diopter change; besides, OK lenses only need to be worn at night, which has less influence on the wearer’s daily work [11]. Some studies have shown that OK lens can effectively improve diopter of myopic patients, but the long-term directly contact of cornea gives constant mechanical pressure to the eyes, which results certain influence on the corneal layer of the patient [12]. Whether the short-term wear has influence on the endothelial cells and corneal thickness of the wearer is unclear.

Therefore, we carried out a study of short-term OK lens or frame glasses wear for myopia correction in juvenile patients with low to moderate myopia, so as to investigate the influence on central corneal thickness and corneal endothelial cells.

## Materials and methods

### Clinical data

The data of 100 adolescents (200 eyes) with low to moderate myopia admitted to Tianjin Eye Hospital from March 2016 to July 2017 were analyzed retrospectively and grouped according to the different correction methods. There were 53 patients in experimental group (28 males and 25 females, aged 8–15 years, with an average age of 11.4 ± 2.2 years) and 47 patients in control group (25 males and 22 females, aged 8–15 years, with an average age of 11.0 ± 2.3 years). This study was approved by the Medical Ethics Committee of Tianjin Eye Hospital. Written informed consent form was obtained from all patients and their guardians.

### Inclusion criteria

Patients were eligible if they were aged 8–15 years old; had diopter from − 1.50 D to − 5.00 D, had with-the-rule astigmatism less than 1.50 D; had against-the-rule astigmatism less than 1.00 D; could have corrected visual acuity of both eyes at 1.0; could master the scientific lenses wear method; had complete clinical data; and cooperated with correction treatment, follow up and regular review.

### Exclusion criteria

Patients were excluded if they had immunological defects, congenital defects, mental diseases, asthma or malignant tumors and needed long-term medication; or were unfit for the correction plans.

### Instruments and materials

Overnight OK contact lenses (Equines II, oxygen permeability 90–110, geometric reverse four-arc surface) applied Corneal Refractive Technology. The slit lamp microscope (YZ5S) was purchased from Topcon, Japan. Corneal topography (TM-4) was purchased from Tomey, Japan. Keratometer (ARK-510A) was purchased from Nidek, Japan. Iolmaster was purchased from Zeiss, Germany. Non-contact tonometer (Canon TX-F) was purchased from Canon, Japan. Corneal endothelial microscope was purchased from Nidek, Japan. VI-Cell XR corneal endothelial cell counter (CEM-530) was purchased from Beckman Coulter, USA.

### Methods

#### Optometry

Visual acuity of patients was tested by a logarithmic visual chart at a distance of 5 m. The uncorrected visual acuity referred to the logarithm of the smallest row that the patient saw (two eyes were tested separately), which was tested starting with pointing the letters from big ones to small ones. A slit lamp was used to examine the patient’s eyes from right to left to see if there was any pathological or morphological change. The nerve nipples, macular area, retinal vessels and retina of patients were examined. During the examination, patients should roll their eyeball to ensure that no relevant diseases occurred. The keratometer and corneal topography were used to detect the overall morphology of the patient’s cornea so as to evaluate the optical characteristics. The tests were carried out for 3 times repeatedly. The number and regularity of corneal endothelial cells were detected by corneal endothelial cell counter.

#### Wear method

The frame glasses in control group were made and wore according to the conventional method, while patients in the experimental group wore the OK lenses for 8–10 h overnight. Eye drop was given after awaking in the morning, and 10 min later the lenses were removed.

### Outcome measures

#### Main outcome measures

Follow-up was performed 3 months, 6 months and 12 months after the start of treatment. The naked-eye vision, diopter, corneal curvature, intraocular pressure, axial length, endothelial cell count and central corneal thickness were examined.

Secondary outcome measures: Complications during the correction period and corneal fluorescein staining grade of patients were observed. See Table 1 for the grading.

**Table 1:** Grading of corneal fluorescein staining [13]

Grade	Degree of injury	Treatment
0	A few dots of staining	No Treatment
I	Increased spots showing a scatter pattern	Continuing lens wear and using a small amount of eye drop for cornea repair until the disappearance of symptoms
II	obvious clinical manifestations, dense and diffuse spots	
III	A large number of stippling stains, aggregation and fusion occurred in a large range	Stopping treatment, adjusting the lens, and giving symptomatic medication until the disappearance of symptoms
IV	Diffuse stippling stains of the whole cornea, mass fusion or even complete epithelial loss	

#### Statistical analysis

In this study, SPSS 20.0 (Shanghai Cabit, China) was used for statistical analyses. GraphPad Prism 7 (Shenzhen SOFTHEAD, China) was used for output of figures. Measurement data were expressed as mean ± standard deviation, compared between groups by independent sample t-test, and compared within groups between different time points by paired t-test. The counting data were expressed as percentage (%), processed by chi-square test. There was a statistical difference when *p <* 0.05.

## Results

### Analysis of clinical data

There was no statistical difference in clinical data between the two groups (all *p >* 0.05), so the two groups were comparable. See Table 2.

**Table 2:** Comparison of clinical data (n, %)

Factor	Experimental group (*n* = 53)	Control group (*n* = 47)	χ^2^	*p*
Gender			0.045	0.832
Male	28 (52.83)	25 (53.19)		
Female	25 (47.17)	22 (46.81)		
Age (year)			0.011	0.916
> 10 years	22 (41.51)	20 (42.55)		
≤ 10 years	31 (58.49)	27 (57.45)		
Course of disease			0.645	0.422
> 6 months	10 (18.87)	12 (25.53)		
≤ 6 months	43 (81.13)	35 (74.47)		
Diopter	3.84 ± 0.92	3.62 ± 0.84	1.758	0.080

### Naked-eye vision and diopter

**Fig. 1:**
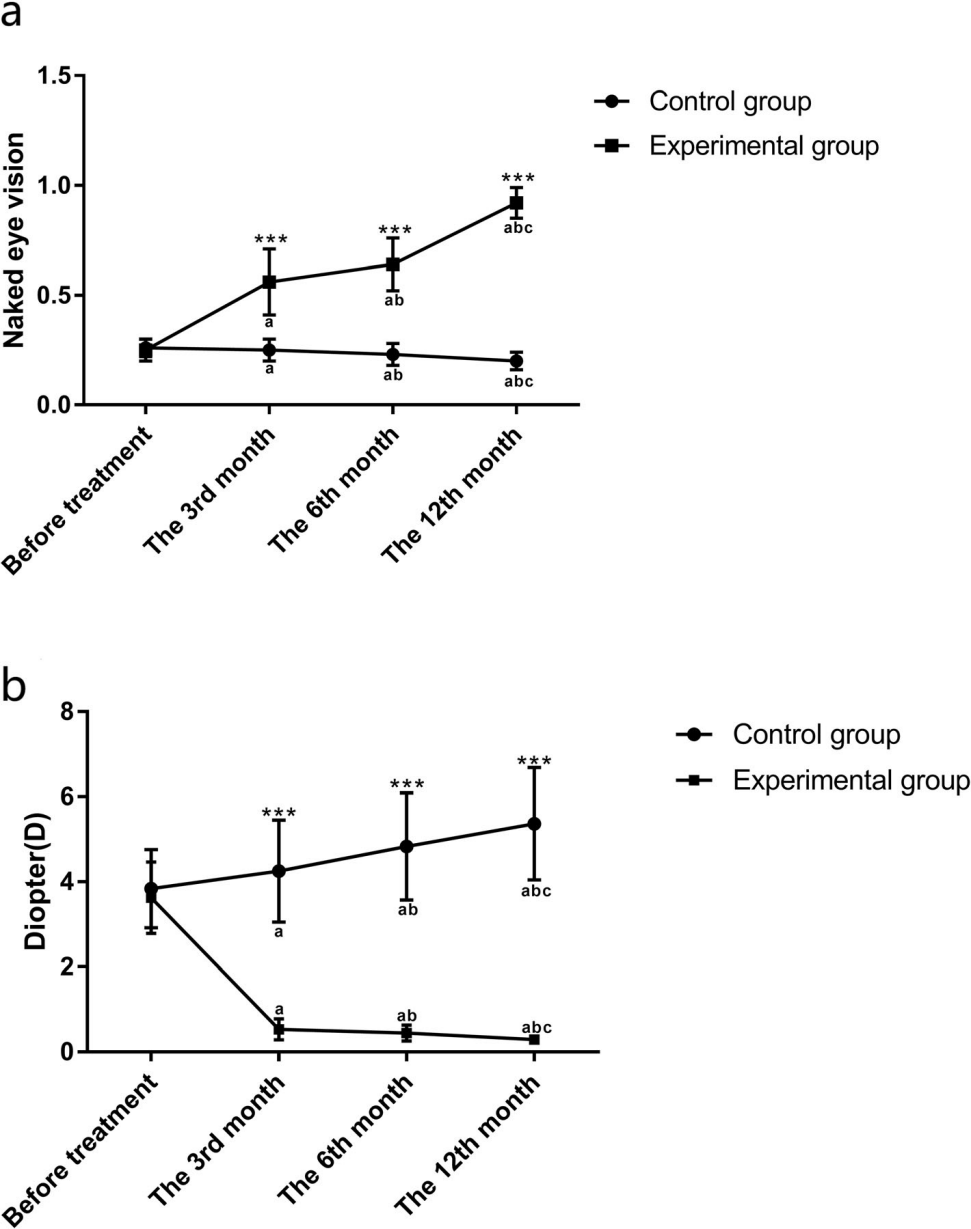
Naked-eye vision and diopter in the two groups. **a**: The naked-eye vision of the control group was significantly lower than that of the experimental group at the 3rd, 6th and 12th months (^***^p < 0.001). **b**: The diopter of the control group was significantly higher than that of the experimental group at the 3rd, 6th and 12th months (^***^p < 0.001). Compared with before treatment, ^a^*p <* 0.01; compared with the 3rd month, ^b^*p <* 0.01; compared with the 6th month, ^c^*p <* 0.01

It was found that the naked-eye vision of patients increased gradually in the experimental group, while decreased gradually in the control group. The naked-eye vision of patients in the experimental group was significantly higher than that in the control group at the 3rd, 6th and 12th months (all *p <* 0.001). The diopter gradually decreased in the experimental group, while gradually increased in the control group, and was significantly lower in the experimental group than in the control group at the 3rd, 6th and 12th months (all *p <* 0.001). There was a difference between any other time points (within group) in both groups in terms of naked-eye vision and diopter (naked-eye vision: *F* = 32.098 in the control group, *F* = 727.005 in the experimental group, both *p <* 0.001; diopter: F = 29.613 in the control group, F = 1344.184 in the experimental group, both *p <* 0.001). See Table 3, Table 4 and Fig. 1.

**Table 3:** Comparison of naked-eye vision

Group	Control group (*n* = 94)	Experimental group (*n* = 106)	t	*p*
Before treatment	0.26 ± 0.04	0.25 ± 0.05	1.569	0.118
The 3rd month	0.25 ± 0.05^a^	0.56 ± 0.15^a^	20.063	0.000
The 6th month	0.23 ± 0.05^ab^	0.64 ± 0.12^ab^	32.176	0.000
The 12th month	0.20 ± 0.04^abc^	0.92 ± 0.07^abc^	90.548	0.000

Compared with before treatment, ^a^*p <* 0.01; compared with the 3rd month, ^b^*p <* 0.01; compared with the 6th month, ^c^*p <* 0.01

**Table 4:** Comparison of diopter (D)

Group	Control group (*n* = 94)	Experimental group (*n* = 106)	t	*p*
Before treatment	3.84 ± 0.92	3.62 ± 0.84	1.758	0.080
The 3rd month	4.25 ± 1.20^a^	0.53 ± 0.25^a^	29.486	0.000
The 6th month	4.83 ± 1.26^ab^	0.44 ± 0.19^ab^	33.435	0.000
The 12th month	5.36 ± 1.32^abc^	0.29 ± 0.08^abc^	37.168	0.000

Compared with before treatment, ^a^*p <* 0.01; compared with the 3rd month, ^b^*p <* 0.01; compared with the 6th month, ^c^*p <* 0.01.

### Corneal curvature, intraocular pressure and axial length during follow-up period

**Fig. 2:**
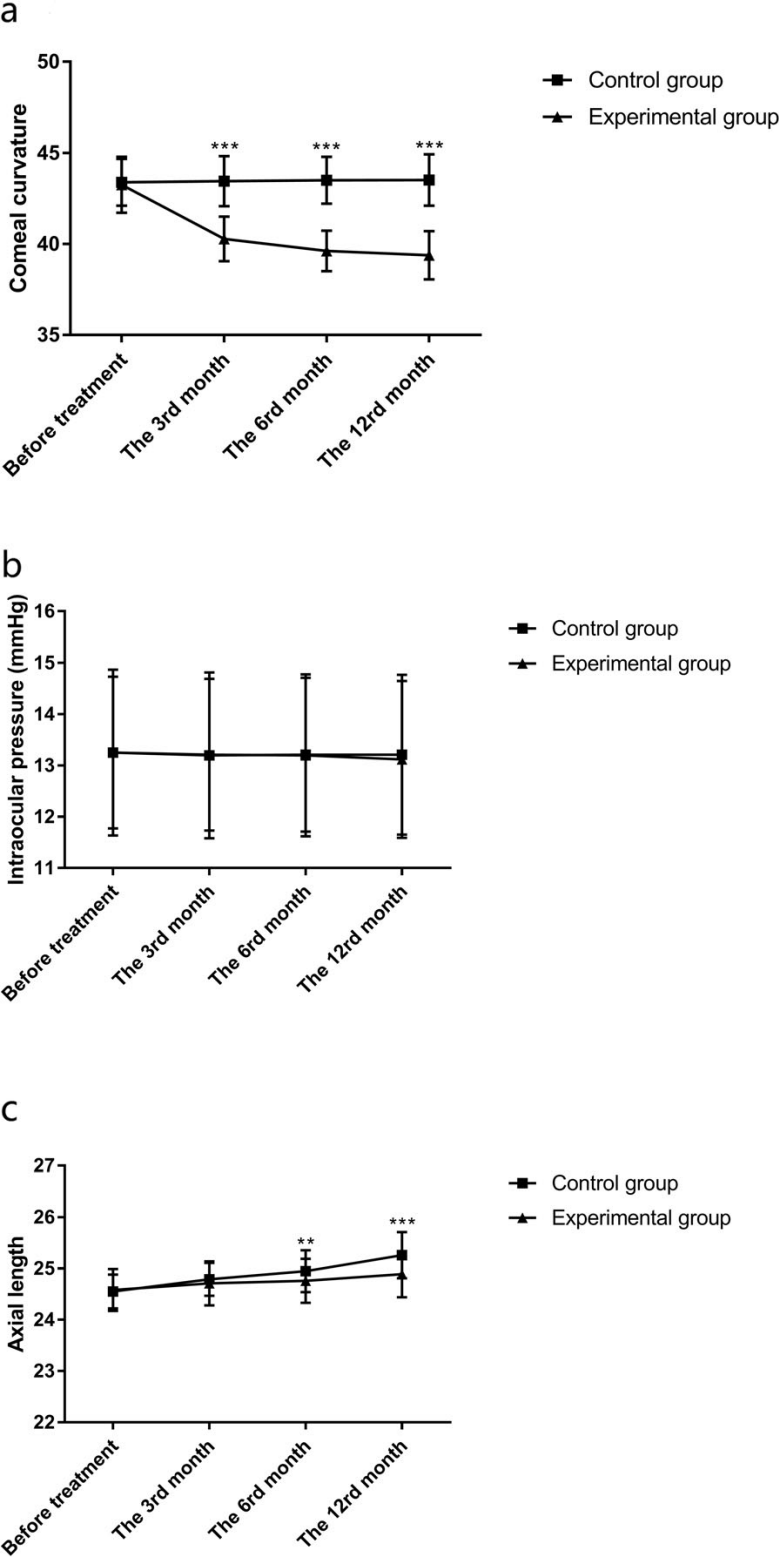
Changes of corneal curvature, intraocular pressure and axial length. **a**: Compared with the control group, the corneal curvature in the experimental group decreased significantly at the 3rd, 6th and 12th months, ^***^p < 0.0001. **b**: There was no statistical difference in intraocular pressure between the two groups at each time point (all *p >* 0.05). **c**: The axial length at the 6th and 12th months in the control group was significantly higher than that in the experimental group, ^**^p < 0.01, ^***^p < 0.001

It was found that the corneal curvature of the experimental group decreased significantly compared with that of the control group at the 3rd, 6th and 12th months of treatment (all *p <* 0.001). The differences of intraocular pressure between the two groups were not statistically significant before treatment as well as at the 6th and 12th months of treatment (all *p >* 0.05). The increases of axial length in the experimental group were significantly less than those in the control group at the 6th and 12th months (*p =* 0.002 and *p <* 0.001). The differences of corneal curvature and intraocular pressure in the control group between each time point were not significant (corneal curvature: F = 0.150, *p =* 0.930; intraocular pressure *F* = 0.019, *p =* 0.996), but there were differences in axial length between each time point (*F* = 8.345, *p <* 0.001). In the experimental group, there was no difference in intraocular pressure and axial length (intraocular pressure *F* = 0.130, *p =* 0.942; axial length: *F* = 1.393, *p =* 0.244), but there were differences in corneal curvature between each time point (*F* = 196.545, *p <* 0.001). See Tables 5, 6, 7, 8 and Fig. 2.

**Table 5:** Changes of corneal curvature (D)

Group	Control group (*n* = 94)	Experimental group (*n* = 106)	t	*p*
Before treatment	43.40 ± 1.30	43.25 ± 1.54	0.747	0.456
The 3rd month	43.45 ± 1.38	40.28 ± 1.23^a^	17.058	0.000
The 6th month	43.50 ± 1.29	39.62 ± 1.12^ab^	22.298	0.000
The 12th month	43.52 ± 1.42	39.38 ± 1.32^ab^	21.268	0.000

Compared with before treatment, ^a^*p <* 0.01; compared with the 3rd month, ^b^*p <* 0.01

**Table 6:** Changes of intraocular pressure (mmHg)

Group	Control group (*n* = 94)	Experimental group (*n* = 106)	t	*p*
Before treatment	13.25 ± 1.48	13.25 ± 1.62	0.000	0.999
The 3rd month	13.20 ± 1.62	13.21 ± 1.48	0.045	0.964
The 6th month	13.21 ± 1.50	13.20 ± 1.58	0.046	0.963
The 12th month	13.21 ± 1.56	13.12 ± 1.53	0.411	0.682

**Table 7:** Changes of axial length (mm)

Group	Control group (*n* = 94)	Experimental group (*n* = 106)	t	*p*
Before treatment	24.55 ± 0.33	24.58 ± 0.41	0.565	0.573
The 3rd month	24.79 ± 0.32^a^	24.71 ± 0.43	1.477	0.141
The 6th month	24.95 ± 0.41^ab^	24.76 ± 0.43	3.188	0.002
The 12th month	25.26 ± 0.45^abc^	24.89 ± 0.45	5.804	0.000

Compared with before treatment, ^a^*p <* 0.01; compared with the 3rd month, ^b^*p <* 0.01; compared with the 6th month, ^c^*p <* 0.01

**Table 8:** Comparison of difference value in intraocular pressure (mmHg)

Group	Control group (*n* = 94)	Experimental group (*n* = 106)	t	*p*
The 3rd month	0.05 ± 0.09	0.04 ± 0.07	0.869	0.386
The 6th month	0.04 ± 0.05	0.05 ± 0.06	1.285	0.200
The 12th month	0.04 ± 0.05	0.03 ± 0.04	1.594	0.123

### Changes of endothelial cell count and central corneal thickness

**Fig. 3:**
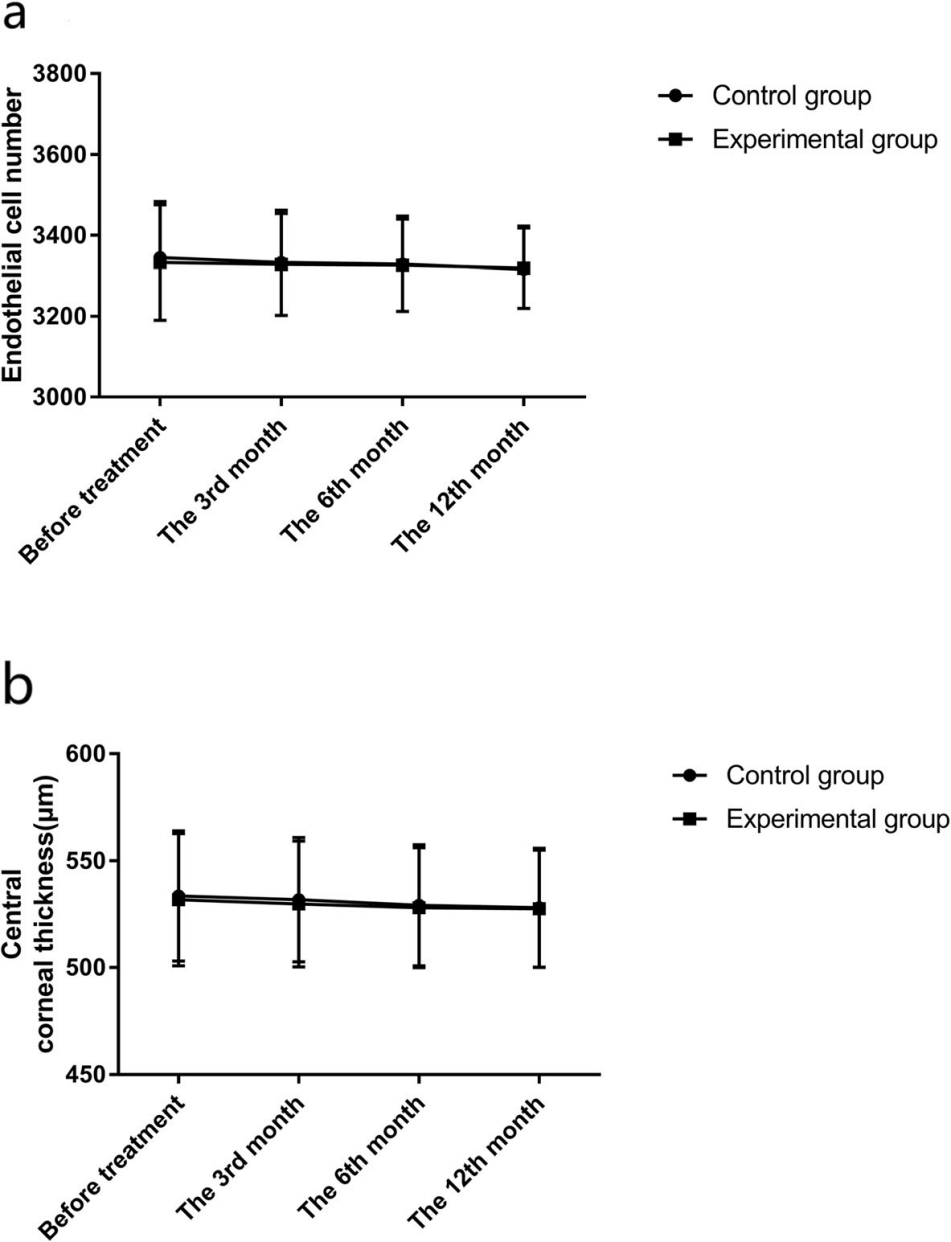
Changes of endothelial cell count and central corneal thickness. **a**: There was no statistical difference in endothelial cell count in both groups between each time point (all *p >* 0.05). **b**: There was no statistical difference in central corneal thickness in both groups between each time point (all *p >* 0.05)

There was no difference in the endothelial cell count and central corneal thickness as well as their difference values between the two groups (all *p >* 0.05). Additionally, the differences of endothelial cell count and central corneal thickness within each group between different time points were also not significant (all *p >* 0.05). See Tables 9, 10, 11, 12 and Fig. 3.

**Table 9:** Changes of endothelial cell count

Group	Control group (*n* = 94)	Experimental group (*n* = 106)	t	*p*
Before treatment	3345.15 ± 138.54	3332.72 ± 142.88	0.624	0.533
The 3rd month	3332.85 ± 129.68	3328.51 ± 126.36	0.239	0.811
The 6th month	3329.27 ± 118.61	3326.39 ± 113.84	0.175	0.862
The 12th month	3316.17 ± 106.51	3318.84 ± 99.74	0.182	0.856

**Table 10:** Comparison of difference value in endothelial cell count

Group	Control group (*n* = 94)	Experimental group (*n* = 106)	t	*p*
The 3rd month	30.08 ± 25.21	28.54 ± 21.76	0.460	0.646
The 6th month	34.68 ± 23.58	30.84 ± 25.42	1.108	0.269
The 12th month	44.48 ± 38.54	40.35 ± 35.84	0.782	0.435

**Table 11:** Changes of central corneal thickness (μm)

Group	Control group (*n* = 94)	Experimental group (*n* = 106)	t	*p*
Before treatment	533.55 ± 30.51	531.85 ± 31.05	0.390	0.697
The 3rd month	531.84 ± 29.18	529.84 ± 29.58	0.481	0.631
The 6th month	529.18 ± 28.39	528.11 ± 28.12	0.267	0.790
The 12th month	528.10 ± 27.92	527.62 ± 27.62	0.122	0.903

**Table 12:** Comparison of difference value in central corneal thickness (μm)

Group	Control group (*n* = 94)	Experimental group (*n* = 106)	t	*p*
The 3rd month	4.92 ± 2.35	4.21 ± 3.12	1.830	0.070
The 6th month	5.34 ± 2.35	4.84 ± 2.12	1.572	0.118
The 12th month	6.36 ± 3.84	6.64 ± 4.15	0.496	0.621

### Complications and corneal fluorescein staining

We found that the incidence of complications in the control group was significantly lower than that in the experimental group (χ^2^ = 4.913, *p =* 0.027), as shown in Table 13. There was no statistical difference in the grade of corneal fluorescein staining between the two groups (Z = − 0.255, *p =* 0.799). See Table 14.

**Table 13:** Comparison of complications (n, %)

Group	Foreign body sensation	Visual abnormality	Aseptic infiltration	Keratitis	Corneal virus infection	Total
Control group (*n* = 47)	0 (0.00)	2 (4.26)	0 (0.00)	1 (2.13)	1 (2.13)	4 (8.51)
Experimental group (*n* = 53)	2 (3.77)	3 (5.66)	1 (1.89)	3 (5.66)	2 (3.77)	11 (20.75)

**Table 14:** Grade of corneal fluorescein staining

Group	0	I	II	III	IV
Control group (*n* = 47)	40 (85.11)	4 (8.51)	1 (2.13)	1 (2.13)	1 (2.13)
Experimental group (n = 53)	44 (83.02)	5 (9.43)	3 (5.66)	1 (1.89)	0 (0.00)

## Discussion

Scholars have suggested that myopia is caused by both genetic and environmental factors, while other studies have shown that long-hour work and reading at short range can also lead to myopia [14–17]. The incidence of myopia among adolescents has increased significantly mainly because their eyeball is in a state of adjustment during long-hour short-range reading. Excessive adjustment causes ciliary muscle to contract continuously, eventually leading to function decline and axial myopia. Survey showed that the incidence of myopia among teenagers is positively related to the increase of school work, and the incidence among adolescents in urban areas is significantly higher than that in rural areas [18]. It is urgent for medical staff to find a better way for myopia treatment and prevention.

OK lens is a kind of auxiliary treatment, which differs from ordinary contact lens in terms of design concept, technology and materials. The OK lens adopts a multi-arc design, which flattens patients’ cornea, reshapes the corneal curvature, improves visual acuity, and avoids the progress of myopia [19]. However, it is not clear whether there is a safety problem when OK lens directly contacts the cornea of patients. Therefore, this study aimed to explore the correction effects of short-term OK lens or ordinary frame glasses wear, and the influence on central corneal thickness and corneal endothelial cells in adolescents with low to moderate myopia to provide a basis for clinicians in terms of correction plans.

Wen’s study showed that the naked-eye vision of juvenile myopia patients was obviously improved and the diopter was reduced by wearing OK lenses [20]. In this study, we compared two groups of patients and found that the naked-eye vision in control group (ordinary frame glasses) was significantly lower than that in the experimental group (OK lenses) at each time point, and the diopter in the control group was significantly increased, with significant differences when comparing with that in the experimental group at each time point. It can be indicated that the OK lens significantly improved the visual acuity and diopter in juvenile patients. Wang’s 6-month study showed that OK lenses wear had no significant impact on patients’ intraocular pressure, and the axial length in patients wearing frame glasses was significantly longer than that in patients wearing OK lenses [21]. We also detected the changes of corneal curvature, axial length and intraocular pressure in the two groups of patients. We found that the corneal curvature in the control group was higher than that in the experimental group at each time point in the correction process. Although the axial length increased in both groups, it was significantly lower in the experimental group than in the control group at the 6th and 12th month of correction. The intraocular pressure between the two groups had no significant difference during the correction process. Our results indicate that OK lenses can improve the corneal curvature and axial length, and have no obvious influence on the intraocular pressure in myopic patients, which is beneficial to the correction of visual acuity. Study of Zhou et al. found that the corneal curvature of patients wearing OK lenses was significantly lower than that of patients wearing frame glasses, which is consistent with our results [22]. We also examined the corneal thickness and corneal endothelial cells in two groups of patients, and found no difference in the two indexes between the two groups. The results indicate that although the OK lens directly contacts patients’ cornea, it has no significant influence on corneal thickness and endothelial cell count. Besides, we found no significant difference in the corneal fluorescein staining grade between the two groups. The incidence of complications in the control group was significantly lower than that in the experimental group. It indicates that patients wearing OK lenses are prone to have complications. But we found that the main cause of complications in the experimental group was the irregular wear method and daily cleaning, and the complications were reduced after giving guidance to the patients.

However, there are still some limitations in this study. Firstly, the follow-up period was not long enough; the mechanism of action has not been explored in depth; indexes such as changes of tear film and peripheral corneal thickness were not analyzed. Secondly, it is not clear whether reading time and/or screen time had any impact on the study results for we failed to record the relevant data. Lastly, we used both eyes from each participant, which may cause confounding in the study results. Therefore, we will carry out further studies with larger sample size, longer follow-up, more adequate detection indexes to explore the mechanism and to verify our results. In recent years, there is a kind of soft eye contact lenses in the market. Report showed the safety and convenience of soft lenses as compared with the rigid lenses [23]. But at present there are no reports about its long-term safety. Therefore, we also hope to delve into this aspect in future studies, so as to provide reference for clinical treatment.

## Conclusions

In summary, short-term OK lens wear can effectively improve the naked-eye vision in adolescents with low to moderate myopia without significant impact on the central corneal thickness and corneal endothelial cells. It is a relatively safe method to correct myopia. And it is necessary to master the correct and scientific optometry as well as methods for cleaning and preservation to better play its role.

## Data Availability

The analysed datasets generated during the study are available from the corresponding author on reasonable request.

## Abbreviation

OK: orthokeratology

## References

[1] Dolgin E (2015). The myopia boom. Nature..

[2] Williams KM, Venturini C, Yonova EH, Hysi PG, Plomin R, Hammond CJ (2015). Evidence for shared genetic factors between myopia and intelligence in the twins early development study (TEDS). Minn Hist.

[3] Hou W, Norton TT, Hyman L, Gwiazda J (2018). Axial elongation in myopic children and its association with myopia progression in the correction of myopia evaluation trial. Eye & contact lens..

[4] Holden BA, Fricke TR, Wilson DA (2016). Global prevalence of myopia and high myopia and temporal trends from 2000 through 2050. Ophthalmology..

[5] Woo R, Akil H, Koulisis N (2017). Olmos de Koo LC, tan JCH. Sustained resolution of macular Retinoschisis after trabeculectomy in a patient with progressive Glaucoma. J Glaucoma.

[6] Kang MT, Li SM, Peng X (2016). Chinese eye exercises and myopia development in school age children: a nested case-control study. Sci Rep.

[7] Scheiman M, Gwiazda J, Zhang Q (2016). Longitudinal changes in corneal curvature and its relationship to axial length in the correction of myopia evaluation trial (COMET) cohort. J Opt.

[8] Morjaria P, Murali K, Evans J, Gilbert C (2016). Spectacle wearing in children randomised to ready-made or custom spectacles, and potential cost savings to programmes: study protocol for a randomised controlled trial. Trials..

[9] Dugel PU, Hillenkamp J, Sivaprasad S, et al. Baseline visual acuity strongly predicts visual acuity gain in patients with diabetic macular edema following anti-vascular endothelial growth factor treatment across trials. Clinical ophthalmology (Auckland, NZ). 2016;10:1103–1110. 10.2147/OPTH.S100764 PMC4913960 27366049

[10] Nelson BA, Gunton KB, Lasker JN, Nelson LB, Drohan LA (2008). The psychosocial aspects of strabismus in teenagers and adults and the impact of surgical correction. Journal of American Association for Pediatric Ophthalmology and Strabismus.

[11] Swarbrick HA, Alharbi A, Watt K, Lum E, Kang P (2015). Myopia control during orthokeratology lens wear in children using a novel study design. Ophthalmology..

[12] Han X, Xu D, Ge W, Wang Z, Li X, Liu W (2018). A comparison of the effects of Orthokeratology Lens, Medcall Lens, and ordinary frame glasses on the accommodative response in myopic children. Eye & contact lens.

[13] Bron AJ, Evans VE, Smith JA (2003). Grading of corneal and conjunctival staining in the context of other dry eye tests. Cornea..

[14] Goldschmidt E, Jacobsen N (2013). Genetic and environmental effects on myopia development and progression. Eye.

[15] Guo L, Yang J, Mai J, Du X, Guo Y, Li P, Yue Y, Tang D, Lu C, Zhang W-H (2016). Prevalence and associated factors of myopia among primary and middle school-aged students: a school-based study in Guangzhou. Eye.

[16] Recko M, Stahl ED (2015). Childhood myopia: epidemiology, risk factors, and prevention. Mo Med.

[17] Rudnicka AR, Kapetanakis VV, Wathern AK (2016). Global variations and time trends in the prevalence of childhood myopia, a systematic review and quantitative meta-analysis: implications for aetiology and early prevention. Br J Ophthalmol.

[18] Epstein D (1984). The correlation between the amplitude of accommodation and low-luminance myopia. Acta Ophthalmol.

[19] Zhang H, Zhao F, Hutchinson DS (2017). Conjunctival microbiome changes associated with soft contact Lens and Orthokeratology Lens wearing. Invest Ophthalmol Vis Sci.

[20] Wen D, Huang J, Chen H (2015). Efficacy and acceptability of Orthokeratology for slowing myopic progression in children: a systematic review and meta-analysis. J Ophthalmol.

[21] Wang X, Zhang L, Qiu Y, et al. Influence of orthokeratology lens on vision,intraocular pressure and biometric measurement parameters of adolescent myopia. Chongqing Medicine 2017.

[22] Zhou ZX, Xu SS, Yi SP (2016). Hospital NT.

[23] Ramakrishnan R, Kadu AD, Naik A (2016). Corneal changes in soft contact lens wearers. Journal of Clinical Ophthalmology and Research.

